# Meckel’s Diverticulum Injuries after Blunt Trauma

**DOI:** 10.3390/jcm13061614

**Published:** 2024-03-12

**Authors:** Piotr T. Arkuszewski, Karol K. Kłosiński, Oliwia J. Kawa, Bartosz M. Czyżewski, Zbigniew W. Pasieka

**Affiliations:** 1 Department of Biomedicine and Experimental Surgery, Faculty of Medicine, Medical University of Lodz, Narutowicza 60, 90-136 Lodz, Poland; piotr.tomasz.arkuszewski@umed.lodz.pl (P.T.A.); zbigniew.pasieka@umed.lodz.pl (Z.W.P.); 2 Faculty of Medicine, Medical University of Lodz, Kościuszki 4, 90-419 Łódź, Poland; oliwia.kawa@stud.umed.lodz.pl (O.J.K.); bartosz.czyzewski@stud.umed.lodz.pl (B.M.C.)

**Keywords:** Meckel’s diverticulum, blunt abdominal trauma, traumatic rupture

## Abstract

**Background**: The complications associated with Meckel’s diverticulum (MD) are well-known. However, blunt injuries regarding MD have not been widely described in the literature. This was the reason for undertaking research on MD lesions. **Materials and Methods**: The materials consisted of 28 cases of damage to MD after blunt trauma published during the years 1921–2022. The collected data were subjected to statistical analysis. **Results**: The following MD injuries have been identified, starting with the most common isolated perforation of MD, bleeding from the area of MD, perforation of MD with concomitant tearing of the mesentery intestines and bleeding, intussusception of MD, and intramural hematoma of MD with adjacent mesenteric hematoma. Most injuries were caused by a traffic accident, followed by sports, accidents at work, and then violence. Almost all the cases (27) involved men. Several possible mechanisms may contribute to post-traumatic damage to MD. First of all, they are associated with abdominal compression and a secondary increase in intra-abdominal pressure as well as with the action of shearing forces during deceleration. **Conclusions**: Traumatic MD injuries are differentiated and very rare. They can coexist with other serious injuries to the abdominal organs.

## 1. Introduction

Meckel’s diverticulum (MD) is a congenital anomaly of the gastrointestinal tract. It is a true diverticulum of the small intestine located in the ileum. MD can contain heterotopic (ectopic) tissue. The most common heterotopic tissue is gastric and pancreatic tissue. However, carcinoid, duodenal tissue, lipoma, mucocele, leiomyoma, metastatic adenocarcinoma, jejunal mucosa, and Brunner’s gland have been found in these diverticula. The majority of MDs are asymptomatic and were discovered incidentally during surgical procedures [1,2,3,4,5].

Complications of MD of a morbid nature have been widely described in the literature. These include the following examples: intestinal obstruction (mesodiverticular band, volvulus, intussusception, persistent vitello-intestinal canal, strangulation, carcinoma), inflammation, hemorrhage, perforation, component of hernial sac, volvulus, umbilical fistula, foreign body, and myxoglobulosis [1,2,3,4,5]. However, the structure can also undergo post-traumatic damage, resulting in serious consequences requiring surgical treatment; these are very few and isolated cases. They were not presented in the form of a collective study, which prompted the authors to take up this topic. An issue related to diverticulum that has not yet been comprehensively examined in the literature is damage to this structure after blunt trauma. This prompted the authors to address this issue by considering multiple cases.

The aim of the study is to describe the damage to MD (and its related structures, such as the blood vessels, mesentery, and loop of the small intestine) following blunt trauma and examine post-traumatic intussusception of the small intestine associated with MD. The analysis examines the nature of the injury itself and its related injuries, and whether they were accompanied by injuries to other abdominal organs. It also relates the occurrence of injury to the personal characteristics of the patients, such as sex and age.

## 2. Materials and Methods

The materials consisted of 28 cases of damage to MD after blunt trauma published during the years 1921–2022 (only complete cases). The desk research method was used, understood as the use of existing data that were described as individual cases by other researchers. The research was monographic, qualitative, and quantitative in nature and was conducted in the form of a detailed description. Individual cases were obtained after searching the following Internet databases: PubMed, ClinicalKey, Academic Search Ultimate (EBSCO), BMJ Journals, Elsevier Journals, Embase, Karger, Oxford Journals, Scopus, Springer, and Wiley Online Library. Some articles were found by Google searches. All searches were performed between July 2021 and December 2022.

Each case of damage to MD was entered into a case card; the data included patient age, sex, circumstances of the injury, and any accompanying surgical or radiological injuries to the abdominal cavity organs. The findings are presented in Table 1.

The results were also subjected to statistical analysis. Briefly, structure indices, arithmetic means, standard deviations, and minimum and maximum values were calculated. Following this, the mean ages were compared using the Mann–Whitney test, and the dependence was tested using the chi^2^ independence test with Yates’ correction to account for the small sample size and reduce errors of approximation. This approach was chosen to ensure the validity of our statistical comparisons despite the limited number of cases in some categories. For four-field arrays and counts lower than three, Fisher’s exact test was used.

## 3. Results

The age of the patients ranged from 6 to 60 years, the mean age was 27.3 ± 15.6 years, and half of the subjects were no more than 24 years old. The largest individual age group was 11–20 years (eight cases; 28.6% of all the patients), and second was 21–30 years (six cases; 21.4%). The smallest age group was 41–50 years (one patient; 3.6%). Twenty-three cases (82.1%) were aged up to 40, and five were over 40 (17.9%). Most cases involved men (27 cases; 96.4%). Most injuries were caused by a traffic accident (sixteen cases, 57.1%), followed by sport (five cases; 17.9%), accidents at work (four cases; 14.3%), and then violence (three cases; 10.7%). In three patients (10.7%), damage to MD was accompanied by damage to the abdominal cavity organ, which required surgical treatment. Rupture of the spleen was recorded in two people (7.1%) and the liver in one person (3.6%).

The most common injury turned out to be isolated perforation of MD (eighteen cases; 64.3%), followed by bleeding from the area of MD (four cases; 14.3%) and perforation of MD with concomitant tearing of the mesentery intestines and bleeding (three cases; 10.7%). Finally, intussusception of MD (two cases; 7.1%) and intramural hematoma of MD with adjacent mesenteric hematoma (one case; 3.6%) were also observed but at low frequencies (Table 2).

No statistically significant relationship was found between the circumstances of the injury and the age of the subjects (*p* > 0.05). It is worth noting, however, that, in patients over 40 years of age, MD damage was significantly more likely to be associated with a traffic accident than those under 40 years of age: 80.0% and 52.2%. However, the younger patients (<40 years of age) had more frequent injuries during sports (21.7%) than those who were over 40 years old (0.0%; Table 3).

In addition, no statistically significant relationship was found between age and the occurrence of accompanying injuries (*p* > 0.05). It should be noted that concomitant injuries were observed only in patients up to 40 years of age (13.0%; Table 4).

No statistically significant relationship was observed between age and diagnosis (*p* > 0.05). It is worth noting, however, that isolated perforation of MD was diagnosed in slightly more than half of the patients aged up to 40 years but in all the patients over 40 years of age (100.0%) (Table 5).

A significant relationship was observed between the circumstances of the injury and the diagnosis (*p* = 0.0153). Bleeding from the area of MD was significantly more common for sports injuries (60.0%) than for traffic injuries (6.2%) and those associated with work or violence (0%). Isolated perforation of MD was significantly more common for injuries at work (100.0%) than traffic injuries (68.8%), sports injuries (40.0%), and violence (33.3%) (Table 6).

No statistically significant relationship was found between age and diagnosis, although the relationship was quite close to significance (*p* > 0.05). It should be noted that isolated perforation of MD was recorded in slightly more than half of the patients under 40 years of age (56.5%) but in all those over 40 years (100.0%). This was found to be significant by the maximum likelihood chi^2^ test (Table 7).

## 4. Discussion

Several possible mechanisms may contribute to post-traumatic damage to Meckel’s diverticulum (MD), particularly its perforation.

Some authors propose that post-traumatic perforation of MD may be caused by increased pressure [9,10,15,29,30,32], the transfer of a sudden increase in intra-abdominal pressure to the intestinal lumen [30], or intra-abdominal pressure [29]; however, one study fails to specify the nature of the pressure increase [9]. In our opinion, MD rupture may be caused by increased intra-abdominal pressure alone, without any accompanying increase in pressure in the intestinal lumen.

The combination of increased pressure in the intestinal lumen and secondary pressure in the MD (e.g., after “pushing” contents from the intestine there) may directly result from either direct pressure on the intestine after impact, e.g., when the intestine is close to the abdominal wall, or compression by other structures of the abdominal cavity following a blow to the abdomen, both of which can be described as an increase in intra-abdominal pressure. In both these situations, the pressure on the MD may increase. When the diverticulum is filled with intestinal content or ectopic tissue, preventing its escape into the lumen of the main axis of the small intestine, the pressure exerted by the contents of MD may rupture the diverticulum from the inside, although the original cause was the external pressure.

Almatri et al. show that increased intra-abdominal pressure is transmitted to the intestinal lumen. They note that, while the base of the diverticulum is wide enough to allow the flow of intestinal contents, it is nevertheless smaller than the diameter of the intestine; this increases the pressure in the diverticulum, resulting in its rupture [30]. Park and Lucas propose that such impact causes a rapid increase in pressure in the lumen of the intestine as a closed-loop phenomenon, with the diverticulum as the weakest point [10].

Similarly, perforation can also occur in response to pressure being exerted on the diverticulum by other structures of the abdominal cavity. Vian Ferreira et al. report a case where crushing trauma led to increased intravisceral pressure, resulting in a rupture in a sensitive area, such as the terminal ileum or Meckel’s diverticulum [29]. In turn, post-traumatic perforation of MD appears to result from the diverticulum being crushed between the anterior abdominal wall and the vertebrae [24].

Bhattarai et al. propose that pressure rupture occurs when the intestinal loop is trapped by the closure of its lumen; the resulting compression increases the internal pressure, causing the intestinal wall to rupture at the weakened site [32]. The authors also mention pressure rupture and crushing as possible mechanisms of small intestine perforation in blunt trauma. Pressure rupture occurs when the lumen of the intestine is closed and compression increases the internal pressure, causing a weak spot to rupture. In contrast, crushing occurs when a direct blow crushes a segment of the intestine against a relatively immobile and rigid structure, such as a vertebra [32]. The mechanism of MD injury after blunt trauma associated with strong abdominal compression is confirmed in the analyzed material. In four cases, the person hit the bicycle handlebar [14,19,30,32]. Two people in cars were held by seat belts [10,15]. One person was struck across the lower abdomen with a one-by-four-inch plank while trying to feed it through a circular saw [8]. In one case, trauma of hypogastrium was a result of compression due to being crushed against a wall by a truck [11].

Another indicated mechanism leading to the perforation of MD in blunt trauma is the action of shearing forces during deceleration [10,15,32]. Referring to damage to the small intestine after blunt trauma, it has been proposed that such shearing forces are generated by sudden deceleration or acceleration, e.g., in a fall from a height or in a traffic accident; in such cases, a sudden stop causes the intestine to move forward, while the constant (fixed) points such as the duodenojejunal flexure and ileocaecal junction take the force and are torn away [32]. In contrast, Almatrfi et al. report that rupture of the MD after blunt abdominal trauma is rare because it is not attached to the ligaments or mesentery, and acceleration or deceleration injury is not expected in such cases [30].

Some authors indicate that ongoing inflammation may increase the likelihood of perforation of MD after blunt trauma as it is believed to weaken the diverticulum wall [9,20,23]. However, it was not possible to verify this hypothesis due to a lack of information regarding postoperative histopathological examination presented in the article. Similarly, it is unclear whether the presence of ectopic tissue in MD increases the risk of its perforation after blunt trauma.

It has been proposed that cases where MD perforation is combined with bleeding indicate energy transfer through the abdominal wall. This also results in the tip of MD becoming detached from its fixation point to the mesentery of the small intestine [14].

Diverticulum rupture may occur through a more complex mechanism. When the apex of the MD is connected by a band of connective tissue to the anterior abdominal wall, a rupture may occur in this area following blunt trauma if the diverticulum moves rapidly in relation to the band, e.g., as a result of increased intra-abdominal pressure or inertia forces at its base. However, this was not described in any of the discussed cases.

In cases involving bleeding from a vessel close to MD, the most likely mechanism of injury, in our opinion, is rupture of the blood vessel as a result of overstretching.

It has also been proposed that, following injury, impaired intestinal motility plays a major role in intussusception, with MD as the leading point [16].

The very small amount of accompanying injuries to the abdominal organs (three cases) indicates a very selective mechanism of injury that leads to damage to this structure.

The statistical analysis shows that a much greater risk of MD injury occurs in people under 40 years of age (only 17.9% of the cases being over 40 years of age). In the age group over 40, injuries resulting from traffic accidents predominate (80%). The remaining cases are accidents at work (20%). However, among the cases in the younger age group under 40, despite the fact that traffic accidents also constitute the majority (52.2%), many of the causes of injuries include accidents while playing sports (21.7%). The remaining cases are accidents at work (13%) and accidents caused by the use of violence (13%). The analyzed material does not describe MD injury as a result of blunt trauma in a person over 60 years of age. The occurrence of such injuries correlates with increased life activity (e.g., while practicing sports) and applies to people undertaking various acts.

The lack of complete data (histopathology results were not provided in all the cases) did not allow us to determine whether the presence of ectopic tissue in MD is a risk factor for post-traumatic MD injury. For the same reason, it has not been possible to determine whether diverticulitis increases the risk of MD perforation after blunt trauma.

## 5. Conclusions

Based on the research and analysis carried out, the following conclusions can be drawn:

1. Injuries of the MD after blunt trauma are extremely infrequent.
2. In the study group, the most common diagnosis was post-traumatic isolated MD perforation, which occurred in more than half of the cases, i.e., *n* = 18; this was also accompanied by mesentery rupture in three other patients.
3. Other diagnoses were significantly more frequent in patients aged up to 40 years (47.8%) than in older patients (0%; *p* = 0.047).
4. A significant relationship was observed between the circumstances of the injury and the diagnosis (*p* = 0.0153). Bleeding from the MD area was significantly more common with injuries in sports (60.0%) than work-related and violent injuries (0%) and traffic injuries (6.2%). Isolated MD perforation was closely associated with work-related injuries (100.0%), followed by traffic injuries (68.8%), sports injuries (40.0%), and violent injuries (33.3%).
5. Abdominal injuries accompanying MD lesions after blunt traumas are occasional (only three out of twenty-eight—10.7%—of the patients had another intra-abdominal injury).

## Figures and Tables

**Table 1:** All the analyzed cases.

Source of Information	Age	Sex	Circumstances of Injury	Accompanying Injuries to the Abdominal Cavity Organs Requiring Surgical Treatment (Not Only Surgical)	Final Diagnosis of Meckel’s Diverticulum Only
Gilruth, 1924 [6]	16	M	sport/play (severe injury to the abdomen while playing with some other boys)	-	traumatic perforation of MD
Gamble, 1941 [7]	16	M	work accident (struck in the abdomen near the umbilicus with the handle of a plow while plowing)	-	traumatic perforation of MD, acute diverticulitis
Cullen& Catanzaro, 1954 [8]	58	M	work accident (strike across the lower abdomen with a one-by-four-inch plank while trying to feed it through a circular saw)	-	traumatic perforation of MD
Łazarkiewicz & Czereda, 1968 [9]	40	F	road traffic accident (circumstances unknown)	-	traumatic perforation of MD
Park & Lucas, 1970 [10]	21	M	road traffic accident (car accident, upper body thrown against the steering wheel while lower abdomen and pelvis were restrained by a seat belt)	-	traumatic perforation of MD, hemorrhage from the mesentery of MD, no peritonitis (the mesentery of the MD was the source of bleeding, and there was a 2 × 3 cm perforation at its tip)
Pape et al., 1985 [11]	56	M	road traffic accident (trauma of hypogastrium as a result of compression due to crush against a wall by a truck)	-	traumatic perforation of MD
McAneny, 1989 [12]	35	M	road traffic accident (strike by a car)	-	intra-abdominal hemorrhage from mesodiverticular band disrupted after blunt trauma (a distinct vascular band extending directly from the mesenteric apex to the diverticulum was identified as the source of the hemorrhage)
Luke et al., 1990 [13]	18	M	violence (injury in a brawl, punches and kicks about the face, head and abdomen)	a tear in the upper pole of the spleen—left undisturbed as it was encased in omentum	intramural hematoma of an MD resulting from blunt abdominal trauma, which presented as a delayed small-intestinal obstruction
Sartorelli et al., 2007 [14]	6	M	road traffic accident (blunt trauma to the abdomen with bicycle handle during a crash)	-	traumatic perforation of MD, bleeding from the small bowel mesentery (an area of bleeding was encountered in the small bowel mesentery where the tip of the MD had been torn from a point of fixation to the mesentery)
Kazemi et al., 2009 [15]	36	M	road traffic accident (head-on car collision in which lower abdomen and pelvis were restrained by a seat belt)	-	traumatic perforation of MD with active bleeding from mesodiverticular rupture (the mesentery of this diverticulum was the major source of intra-abdominal bleeding, and the ileal rupture was the cause of contamination)
Benjelloun et al., 2009 [16]	28	M	violence (a hit in the left side of the abdomen with a fist)	-	ileo-ileal intussusception with MD as a lead point
Woodfield et al., 2011 [17]	16	M	sport/play (softball injury after diving for third base and then again for last base)	-	traumatic avulsion medium-sized artery supplying MD (the bleeding point was identified as an avulsed medium-sized artery arising from the small bowel mesentery and supplying a large MD)
Wilson &Dubinsky, 2012 [18]	17	M	road traffic accident (high-speed motor vehicle collision)	grade 5 liver laceration	traumatic intussusception of MD
Mishra et al., 2014 [19]	10	M	road traffic accident (blunt trauma to left lower abdomen with bicycle handle)	-	traumatic perforation of MD
Ekwunife et al., 2014 [20]	29	M	road traffic accident (a front-seat passenger in a vehicle that had burst a tire and subsequently hit a tree; he had not been wearing a seat belt, and his chest and abdomen hit the dashboard)	-	traumatic perforation of MD
Makwana & Hammond, 2015 [21]	20	M	sport/play (punch around the umbilicus during a boxing match)	a grade 3 splenic laceration without active bleeding	traumatic rupture of MD’s blood supply
Batista et al., 2015 [22]	32	M	road traffic accident (car crash)	-	traumatic perforation of the MD
Banu et al., 2015 [23]	8	M	road traffic accident (fall from the bicycle)	-	traumatic perforation of the MD
Tummers et al., 2015 [24]	17	M	sport/play (a firm kick in the abdomen during a soccer game)	-	traumatic perforation of the MD
Ikram & Mohamed, 2016 [25]	43	M	road traffic accident (pedestrian hit by a car)	-	traumatic perforation of the MD
Jayakumar et al., 2017 [26]	23	M	violence (assault by a person using bare hands)	-	traumatic perforation of the MD
Chowdhury et al., 2018 [27]	12	M	sport/play (football)	-	traumatic rupture of MD’s blood supply (during the laparotomy, an injured MD was identified as the source of the significant arterial bleeding)
Lim et al., 2018 [28]	25	M	work accident (bumping into the corner of a machine)	-	traumatic perforation of MD that presented as a chronic enterocutaneous fistula
Ferreira et al., 2019 [29]	36	M	work accident (rollover accident—abdomen stuck underneath the structure of an industrial tractor)	-	traumatic perforation of the MD, rupture of the mesentery with hemorrhage by a distal branch of the superior mesenteric artery (an MD rupture, with a transverse lesion of the distal ileum mesentery and an active hemorrhage by a distal branch of the superior mesenteric artery)
Almatrfi et al., 2020 [30]	9	M	road traffic accident (fall over bicycle’s handlebar)	-	traumatic perforation of the MD
Kojima et al., 2020 [31]	52	M	road traffic accident (motorcyclist collided with a guardrail)	-	traumatic perforation of the MD
Bhattarai et al., 2021 [32]	60	M	road traffic accident (falling from the bike, trauma to the abdomen from the bike’s handle)	-	traumatic perforation of the MD
Martínez-Mardones et al., 2021 [33]	26	M	road traffic accident (motorbike accident)	-	traumatic perforation of MD

**Table 2:** The nature of the damage to MD.

Diagnosis	Number	%
Isolated perforation of MD	18	64.3
Bleeding from the area of MD	4	14.3
MD perforation and accompanying mesenteric rupture with bleeding	3	10.7
MD intussusception	2	7.1
Intramural hematoma of MD with adjacent mesenteric hematoma	1	3.6
Total	28	100.0

**Table 3:** The relationship between age and the circumstances of the injury.

Circumstances of Injury	Age (Years)				Total
	0–40		Above 40		
	*n*	%	*n*	%	
Traffic accident	12	52.2	4	80.0	16
Injuries while playing sports	5	21.7	-	-	5
Accidents at work	3	13.0	1	20.0	4
Accidents caused by the use of violence	3	13.0	-	-	3
Total	23	100.0	5	100.0	28

chi^2^ = 2.435; *p* = 0.487.

**Table 4:** The relationship between age and the occurrence of accompanying injuries.

Occurrence of Accompanying Injuries to the Abdominal Organs	Age (Years)				Total
	0–40		Above 40		
	*n*	%	*n*	%	
Yes	3	13.0	-	-	3
No	20	87.0	5	100.0	25
Total	23	100.0	5	100.0	28

chi^2^ = 0.730; *p* = 0.393.

**Table 5:** The relationship between age and the diagnosis.

Diagnosis	Age (Years)				Total
	0–40		Above 40		
	*n*	%	*n*	%	
Isolated perforation of MD	13	56.6	5	100.0	18
Bleeding from the area of MD	4	17.4	-	-	4
MD perforation and accompanying mesenteric rupture with bleeding	3	13.0	-	-	3
MD intussusception	2	8.7	-	-	2
Intramural hematoma of MD with adjacent mesenteric hematoma	1	4.3	-	-	1
Total	23	100.0	5	100.0	28

chi^2^ = 3.382; *p* = 0.496.

**Table 6:** Relationship between the circumstances of the injury and the diagnosis.

Diagnosis	Circumstances of Injury							
	Traffic Accident		Injuries While Playing Sports		Accidents at Work		Accidents Caused by the Use of Violence	
	*n*	%	*n*	%	*n*	%	*n*	%
Isolated perforation of MD	11	68.8	2	40.0	4	100.0	1	33.3
Bleeding from the area of MD	1	6.2	3	60.0	-	-	-	-
MD perforation and accompanying mesenteric rupture with bleeding	3	18.8	-	-	-	-	-	-
MD intussusception	1	6.2	-	-	-	-	1	33.3
Intramural hematoma of MD with adjacent mesenteric hematoma	-	-	-	-	-	-	1	33.3
Total	16	100.0	5	100.0	4	100.0	3	100.0

chi^2^ = 24.912; *p* = 0.0153.

**Table 7:** The relationship between the age of the respondents and the diagnosis.

Diagnosis	Age (Years)				Total
	0–40		Over 40		
	*n*	%	*n*	%	
Isolated perforation of MD	13	56.5	5	100.0	18
Other (bleeding from the area of MD; MD perforation and accompanying mesenteric rupture with bleeding; MD intussusception; intramural hematoma of MD with adjacent mesenteric hematoma)	10	43.5	-	-	10
Total	23	100.0	5	100.0	28

chi^2^ = 3.382; *p* = 0.066.

## Data Availability

The data presented in this study are available on request from the corresponding authors.
